# Gas Exchange Rates Decrease and Leaf Temperature Increases in *Nicotiana benthamiana* Leaves Transiently Overexpressing Hemagglutinin in an *Agrobacterium*-Assisted Viral Vector System

**DOI:** 10.3389/fpls.2018.01315

**Published:** 2018-09-04

**Authors:** Ryo Matsuda, Akihiro Ueno, Hirofumi Nakaigawa, Kazuhiro Fujiwara

**Affiliations:** ^1^Department of Biological and Environmental Engineering, Graduate School of Agricultural and Life Sciences, The University of Tokyo, Tokyo, Japan; ^2^Faculty of Agriculture, The University of Tokyo, Tokyo, Japan

**Keywords:** plant-made pharmaceuticals, influenza vaccine antigen, photosynthesis, transpiration, stomatal conductance, chlorophyll fluorescence

## Abstract

In this study, gas exchange characteristics and temperature of *Nicotiana benthamiana* leaves transiently overexpressing hemagglutinin (HA), an influenza vaccine antigen, with an *Agrobacterium tumefaciens*-assisted viral vector were investigated. Inoculation of leaves with an empty viral vector not containing the HA gene decreased the net photosynthetic rate (*P*_n_) and transpiration rate (*T*) from 2 to 3 days post-infiltration (DPI) in the *A. tumefaciens* suspension. Expression of HA with the vector decreased *P*_n_ and *T* to much lower levels until 4 DPI. Such significant decreases were not observed in leaves infiltrated with suspension of *A. tumefaciens* not carrying the viral vector or in uninfiltrated leaves. Thus, viral vector inoculation itself decreased *P*_n_ and *T* to a certain extent and the HA expression further decreased them. The decreases in *P*_n_ and *T* in empty vector-inoculated and HA expression vector-inoculated leaves were associated with decreases in stomatal conductance, suggesting that the reduction of gas exchange rates was caused at least in part by stomatal closure. More detailed gas exchange and chlorophyll fluorescence analyses revealed that in HA vector-inoculated leaves, the capacity of ribulose-1,5-bisphosphate carboxylase/oxygenase to assimilate CO_2_ and the capacity of photosynthetic electron transport *in planta* were downregulated, which contributed also to the decrease in *P*_n_. Leaf temperature (LT) increased in viral vector-inoculated leaves, which was associated with the decrease in *T*. When HA vector-inoculated leaves were grown at air temperatures (ATs) of 21, 23, and 26°C post-infiltration, HA accumulated earlier in leaves and the days required for HA content to attain its peak became shorter, as AT was higher. The highest LT was found 1–2 days earlier than the highest leaf HA content under all post-infiltration AT conditions. This phenomenon could be applicable in a non-destructive technique to detect the optimum harvesting date for individual plants to determine the day when leaf HA content reaches its maximum level, irrespective of spatiotemporal variation of AT, in a plant growth facility.

## Introduction

Plants have become a viable platform for producing recombinant proteins such as biopharmaceuticals. Compared with transgenic animal-based or mammalian cell culture-based systems, plant-based systems offer advantages for biopharmaceutical production that include low production cost, high production scalability, and minimum risk of contamination with human pathogens, whereas animal and plant cells have a similar eukaryotic protein synthesis pathway (Fischer and Emans, 2000; Ma et al., 2003; Twyman et al., 2003; Desai et al., 2010). Indeed, two plant-made biopharmaceuticals, taliglucerase alfa (ELELYSO^®^) and interferon-*α* (Interberry *α*^®^), have been approved for human and animal use, respectively, and are already available in the market (Drake et al., 2017). In addition, a seasonal quadrivalent influenza vaccine was recently subjected to a Phase 3 efficacy study in multiple countries^1^ and several other candidates have shown their initial safety and efficacy in early-phase clinical trials (Fischer et al., 2012). There are two main biotechnologies for transgene expression in plants: stable transformation of the nuclear or chloroplast genome and transient gene expression using plant viral or non-viral vectors. Among them, transient gene overexpression in *Nicotiana* species such as *Nicotiana benthamiana* with viral vectors enables rapid mass production of recombinant proteins (Pogue et al., 2002, 2010; Gleba et al., 2004, 2007; Lico et al., 2008; Matoba et al., 2011; Chen et al., 2013; Peyret and Lomonossoff, 2015). The accumulation level of a target recombinant protein using this technology is reported to reach as high as 40% of total soluble protein (Marillonnet et al., 2005) or 3–4 g kg^-1^ on a leaf fresh mass (FM) basis (Marillonnet et al., 2005; Hamorsky et al., 2015) within days post-vector inoculation.

It is expected that massive synthesis and accumulation of a foreign protein in leaves would affect the physiology of the plant. Recent studies have shown that transient overexpression of a recombinant protein using a viral vector could cause severe stress, such as the endoplasmic reticulum (ER) stress, to *N. benthamiana* plants, leading to reduction in protein yield (Hamorsky et al., 2015; Roychowdhury et al., 2018). Viral vector inoculation would also induce defensive responses of plants, including the hypersensitive response (Lam et al., 2001; Mandadi and Scholthof, 2013) and systemic acquired resistance (Fu and Dong, 2013), which affect plant physiology. Understanding the physiological changes caused by viral vector inoculation and recombinant protein expression in host plants can contribute to improving plant conditions that are favorable for recombinant protein production. However, compared to biotechnology for target protein expression or efficacy of target proteins as biopharmaceuticals, less attention has been paid to physiological aspects of plants in plant-based biopharmaceutical production.

Gas exchange, i.e., CO_2_ and water vapor transport driven by photosynthesis, respiration, and transpiration, is a fundamental physiological process in leaves, and is involved in supplying the energy and compounds required for various metabolisms. Given that virus infection often impairs photosynthetic activity and stomatal opening (Chaerle and Van Der Straeten, 2001; Rolfe and Scholes, 2010; Grimmer et al., 2012), such negative effects may be observed in plants inoculated with a virus-derived vector for transient gene expression. Massive synthesis and accumulation of a foreign protein in plant cells can also alter the composition of endogenous proteins, such as significant reduction of ribulose-1,5-bisphosphate carboxylase/oxygenase (RuBisCO; Marillonnet et al., 2004; Bally et al., 2009), which is the key enzyme for photosynthetic CO_2_ assimilation. In addition, stomatal malfunction is expected to influence thermal conditions and temperature of leaves via alteration of latent heat dissipation by transpiration. However, to the best of our knowledge, no study has been undertaken to examine the effects of transient overexpression of biopharmaceutical proteins using viral vectors on gas exchange characteristics of leaves.

The objective of the present study was to reveal gas exchange characteristics and temperature of *N. benthamiana* leaves transiently overexpressing hemagglutinin (HA), an influenza vaccine antigen, in an *Agrobacterium tumefaciens*-assisted viral vector system. We performed a series of experiments. In experiments (Exp.) 1 and 2, time course of net photosynthetic rate (*P*_n_), transpiration rate (*T*), stomatal conductance (*g*_s_), and leaf temperature (LT) in leaves post-inoculation of the viral vector were examined. Post-inoculation plant growth was also evaluated. In Exp. 3, photosynthetic gas exchange characteristics and electron transport capacity of photosystem II (PSII) were analyzed in detail. In Exp. 4, changes in leaf HA content and LT were investigated under different air temperature (AT) conditions.

## Materials and Methods

### Plant Material and Growth Conditions

Seeds of *N. benthamiana* were sown into rockwool cubes (AO36/40, ROCKWOOL B.V., Roermond, Netherlands) and seedlings were grown in a growth room. Light was provided by white fluorescent lamps for 16 h d^-1^ at a photosynthetic photon flux density (PPFD) of 200 μmol m^-2^ s^-1^ at the surface of the rockwool cubes. ATs were 25/20°C (day/night) and mean relative humidity was 30–70%. CO_2_ concentration was not lower than an atmospheric level of 400 μmol mol^-1^ even during the day. The rockwool cubes were subirrigated with tap water for the first week and subsequently with a nutrient solution (prescription A, OAT Agrio Co., Ltd., Tokyo, Japan) at an electrical conductivity of 0.18 S m^-1^ and pH of 6. At 14 days after sowing, seedlings with the second true leaf approximately 3 mm in length were transplanted onto rockwool blocks (Delta 6.5G, ROCKWOOL B.V.) and grown further in the same room until 35, 36, or 38 days after sowing (depending on experiment) at which time they were subjected to the treatments described below. The rockwool blocks were subirrigated with the above nutrient solution. Lateral shoots and flower buds were removed once a week.

### Vector Construction and Agroinfiltration

A “deconstructed” tobamoviral replicon system (Marillonnet et al., 2004, 2005) [magnICON^®^, ICON Genetics GmbH, Halle (Saale), Germany] was used to overexpress HA in *N. benthamiana*. Vector construction and *A. tumefaciens* (GV3101::pMP90, Koncz and Schell, 1986) transformation have been described previously (Matsuda et al., 2012, 2017a). Briefly, the HA construct was designed to target the ectodomain of HA derived from influenza A virus (subtype H1N1, strain A/California/07/2009) to the ER, using an N-terminal secretory signal peptide and a C-terminal HDEL ER-retention signal peptide. The construct was subcloned into the plasmid vector (pICH26212), which contained genes encoding tobamovirus-derived RNA-dependent RNA polymerase and movement protein, to form the HA expression vector (pNM216). Suspension of *A. tumefaciens* was infiltrated into leaves for inoculation, as described previously (Matsuda et al., 2012, 2018). The optical density of the bacterial suspension at 600 nm was 0.03.

### Treatments

In Exp. 1, plants were inoculated with the HA expression vector (pNM216) or an empty vector without the HA gene (pICH26212) by vacuum infiltration of *A. tumefaciens* suspension at a gage pressure of -85 kPa for 2 min or were uninfiltrated. In Exp. 2, plants were inoculated with either of the two vectors or with a “mock” inoculum, i.e., infiltrated with a suspension of *A. tumefaciens* not carrying any plasmid vectors. In Exp. 3, the 8th or 9th oldest leaf from each plant was used. One half of the leaf lamina bordering the primary vein was inoculated with the HA vector by syringe infiltration. The other half was uninfiltrated. In Exp. 1–3, plants were thereafter grown in growth chambers. Light was provided by white light-emitting diodes (LEDs) for 16 h d^-1^ at 200 μmol m^-2^ s^-1^ PPFD, which was measured and adjusted at the tops of plants. AT was maintained at 22 ± 1°C throughout the day. Mean relative humidity was 55–80%. The chambers were ventilated with an air pump at an air exchange rate of approximately 0.5 h^-1^ to keep CO_2_ concentration close to the atmospheric level. In Exp. 4, plants were inoculated with the HA vector or were uninfiltrated. Plants were thereafter grown in the growth chambers under the same light environment at an AT of 21, 23, or 26°C throughout the day. In Exp. 4, experiments were replicated twice for each AT treatment.

### Gas Exchange Measurements

In Exp. 1–3, gas exchange rates of the 8th or 9th oldest leaves were measured using a portable gas-exchange measurement system (LI-6400XT, LI-COR Inc., Lincoln, NE, United States). Light was provided by white LEDs (NSPW310DS, Nichia Corp., Tokushima, Japan). In Exp. 1 and 2, measurements were taken at a PPFD of 200 μmol m^-2^ s^-1^ and an atmospheric CO_2_ concentration (*C*_a_) of 400 μmol mol ^-1^. In Exp. 3, measurements were taken at PPFDs of 250 and 1,200 μmol m^-2^ s^-1^ and at mean *C*_a_ of 98, 194, 292, 390, 589, 787, 987, 1,186, and 1,784 μmol mol^-1^. The coefficient of variation of *C*_a_ was within 1.2%, irrespective of the *C*_a_ level. In all measurements, the leaf chamber was maintained at an AT equal to that for plant growth and a leaf-to-air vapor pressure deficit lower than 1.1 kPa. The air change rate of the leaf chamber was regulated at 0.2 s^-1^. Gas exchange parameters including intercellular CO_2_ concentration (*C*_i_) were calculated based on methods described by von Caemmerer and Farquhar (1981).

### Chlorophyll Fluorescence Measurements

In Exp. 3, chlorophyll (Chl) fluorescence levels of the 9th oldest leaves were measured using a Chl fluorescence measuring system (DUAL-PAM/F, Heinz Walz GmbH, Effeltrich, Germany). The fiber optics were connected to the leaf chamber of the gas exchange measurement system via an adaptor (6400-06, LI-COR Inc.). Chl fluorescence levels and gas exchange rates were measured simultaneously under the conditions described above. Before measurements were taken, leaves were maintained in the dark for at least 30 min. The steady-state and the maximum Chl fluorescence levels in the dark (*F*_o_ and *F*_m_, respectively) were first measured, and subsequently those in the light (*F* and *F*_m_′, respectively) were measured at actinic-light PPFDs of 250 and 1,200 μmol m^-2^ s^-1^. The maximum photochemical quantum yield (*F*_v_/*F*_m_) and effective photochemical quantum yield (*Y*_II_) of PSII were calculated as (*F*_m_ – *F*_o_)/*F*_m_ (Kitajima and Butler, 1975) and (*F*_m_′ – *F*)/*F*_m_′ (Genty et al., 1989), respectively.

### Temperature Measurements

In Exp. 1, 2, and 4, LT of the 8th or 9th oldest leaf of each plant was measured by continuously attaching the measuring junction of a calibrated type-K thermocouple (0.1 mm in diameter) to the leaf surface. AT in each growth chamber was measured using a calibrated type-T thermocouple. Data were collected and recorded every 30 s by a data logger (GL220, Graphtec Corp., Kanagawa, Japan). LT was evaluated as daily mean difference between LT and AT (LT – AT), which was calculated as the averaged difference between LT and AT over a 16-h photoperiod.

### Microscopic Observation of Leaf Epidermis

For uninfiltrated plants and plants inoculated with the HA expression vector, replicas of the adaxial and abaxial epidermis of the 8th or 9th oldest leaves were created at 6 DPI using a liquid adhesive. The replicas were observed under a digital microscope (VHX-1000, Keyence Corp., Osaka, Japan) at 400-times magnification.

### Growth Analysis

Uninfiltrated plants and plants inoculated with the empty vector and the HA expression vector were destructively harvested at 6 days post-infiltration (DPI). Plants at 0 DPI were also harvested. Total leaves of each plant were oven-dried at 100°C for 1 h followed by 80°C for 3 days to determine dry mass (DM).

### Hemagglutinin Quantification

In Exp. 4, the 8th or 9th oldest leaves of plants inoculated with the HA vector were destructively harvested at different DPI. Each leaf lamina was divided into two portions along the primary vein. One half of the leaf lamina was weighed to determine FM and subsequently subjected to HA quantification by sandwich enzyme-linked immunosorbent assay using a commercial kit (Sino Biological Inc., Beijing, China) as previously described by Fujiuchi et al. (2016b). The other half was weighed to determine FM and subsequently oven-dried as described above to determine DM. The ratio of DM to FM was used to convert HA content per unit FM into that per unit DM. Because the absolute HA levels tended to be different depending on the lot number of the kit, HA content was normalized against the maximum level in each AT treatment and replication for evaluation.

### Statistical Analyses

In each experiment, individual plants were considered independent biological replicates. Numbers of replicates per treatment were as follows: 3 in Exp. 1 and 2, 3–4 in Exp. 3, and 4 for HA content and 6 for LT in Exp. 4, respectively. Significant differences between two means and among three means were tested by Welch’s *t* test and Tukey–Kramer’s HSD test, respectively, at *P* < 0.05 using statistical software (R 3.4.3, R Core Team, 2017).

## Results

### Changes in Gas Exchange Rates and Leaf Temperature

**FIGURE 1:**
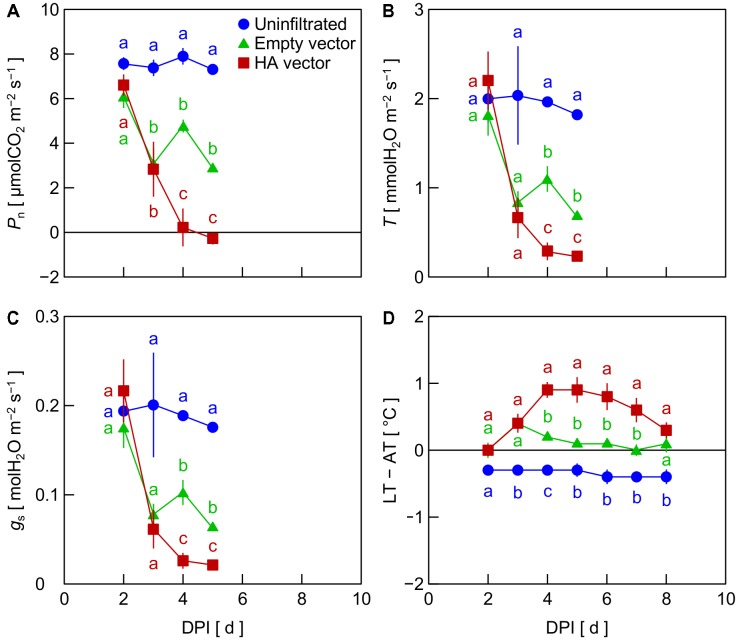
Time course of net photosynthetic rate (*P*_n_, **A**), transpiration rate (*T*, **B**), stomatal conductance (*g*_s_, **C**), and leaf and air temperature difference (LT – AT, **D**) in leaves of *Nicotiana benthamiana* plants at different days post-infiltration (DPI) (Exp. 1). Plants were uninfiltrated or inoculated with the empty vector or the hemagglutinin (HA) expression vector. Data represent means with standard errors of the means (*n* = 3). Means with different lowercase letters at each DPI are significantly different by Tukey–Kramer’s HSD test at *P* < 0.05.

In Exp. 1, *P*_n_ decreased in leaves inoculated with the empty vector and HA vector from 2 DPI, whereas *P*_n_ in uninfiltrated leaves was almost unchanged (**Figure 1A**). *P*_n_ in HA vector-inoculated leaves was significantly lower than that in empty vector-inoculated leaves at 4 and 5 DPI. Similar trends among treatments were observed in *T* and *g*_s_ (**Figures 1B,C**). LT – AT in HA vector-inoculated leaves increased from 2 to 4 DPI and that in empty vector-inoculated leaves slightly increased from 2 to 3 DPI (**Figure 1D**). LT – AT in those leaves thereafter gradually decreased. LT – AT in uninfiltrated leaves was almost constant between 2 and 8 DPI. LT – AT at 4–6 DPI in HA vector- and empty vector-inoculated leaves was approximately 1.2 and 0.4°C higher, respectively, than that in uninfiltrated leaves.

**FIGURE 2:**
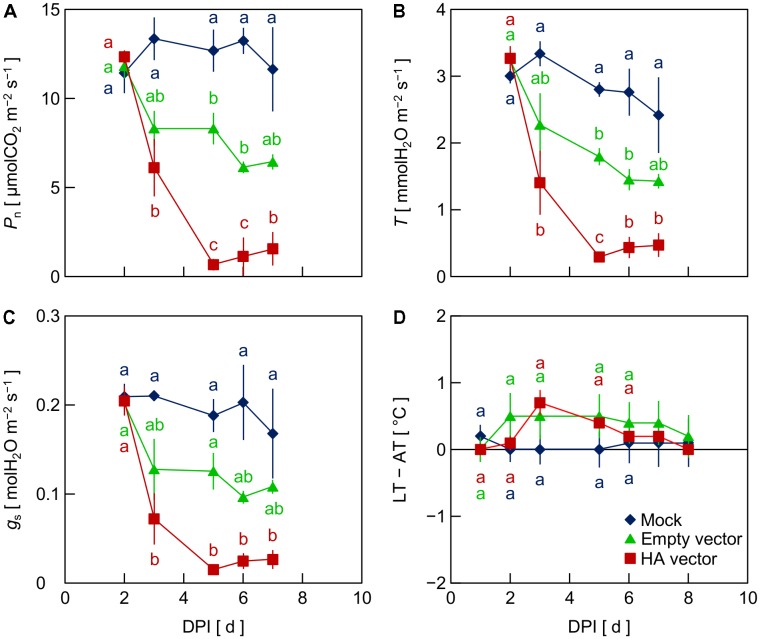
Time course of net photosynthetic rate (*P*_n_, **A**), transpiration rate (*T*, **B**), stomatal conductance (*g*_s_, **C**), and leaf and air temperature difference (LT – AT, **D**) in leaves of *Nicotiana benthamiana* plants at different days post-infiltration (DPI) (Exp. 2). Plants were infiltrated with a mock suspension or inoculated with the empty vector or the hemagglutinin (HA) expression vector. Data represent means with standard errors of the means (*n* = 3). Means with different lowercase letters at each DPI are significantly different by Tukey–Kramer’s HSD test at *P* < 0.05.

To investigate the factor that generated the reduction of *P*_n_, *T*, and *g*_s_ in leaves inoculated with the viral vectors, i.e., the empty and HA vectors, in Exp. 2, we compared mock-inoculated leaves, which were infiltrated with the suspension of *A. tumefaciens* not carrying any plasmid vectors, with empty vector- and HA vector-inoculated leaves. *P*_n_, *T*, and *g*_s_ in mock-inoculated leaves did not markedly decrease throughout the experimental period, as did empty vector- and HA vector-inoculated leaves (**Figures 2A–C**). This indicates that the reduction of *P*_n_, *T*, and *g*_s_ in leaves inoculated with the viral vectors did not result from the vacuum infiltration treatment with an *A. tumefaciens* suspension. In leaves inoculated with the empty and HA vectors, *P*_n_, *T*, and *g*_s_ decreased from 2 to 3 or 4 DPI, being lower in HA vector-inoculated leaves than in empty vector-inoculated leaves (**Figures 2A–C**), as was observed in Exp. 1. LT – AT in empty vector- and HA vector-inoculated leaves tended to be higher than that in mock-inoculated leaves at 3–5 DPI, although the difference was not significant in this experiment partly due to a relatively large variation among biological replicates in empty vector-inoculated leaves (**Figure 2D**).

*g*_s_ is an index of the diffusivity of CO_2_ and water vapor through the stomata and is largely influenced by stomatal density and stomatal opening. We made replicas of the adaxial and abaxial epidermis of uninfiltrated leaves and HA vector-inoculated leaves at 6 DPI and observed them with a digital microscope. There was no notable difference in stomatal density or the shape of guard cells between leaves (data not shown), suggesting that the lower *g*_s_ in HA vector-inoculated leaves was due to impairment of stomatal opening.

### Photosynthetic Characteristics

**FIGURE 3:**
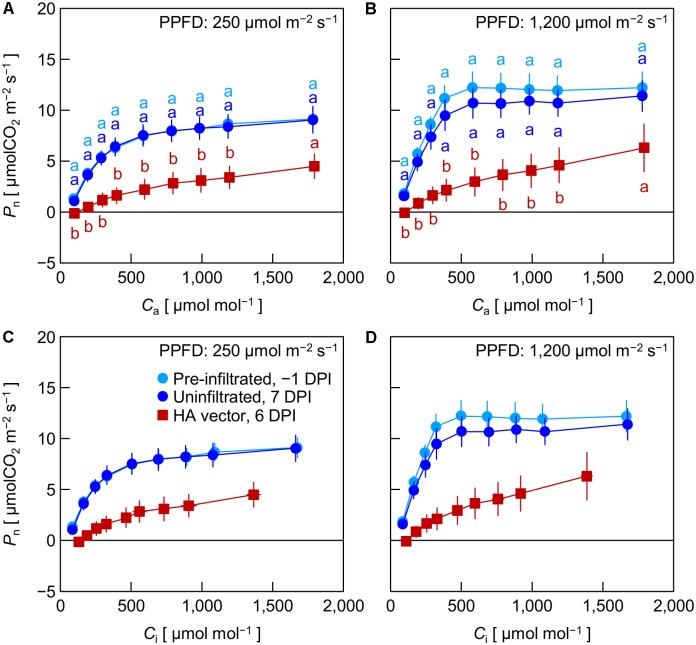
Net photosynthetic rate (*P*_n_) plotted against atmospheric CO_2_ concentration (*C*_a_, **A,B**) or intercellular CO_2_ concentration (*C*_i_, **C,D**) in leaves of *Nicotiana benthamiana* plants (Exp. 3). Measurements were taken at photosynthetic photon flux densities (PPFDs) of 250 and 1,200 μmol m^-2^ s^-1^ at 1 day pre-infiltration (-1 DPI), 7 days post-infiltration (7 DPI) for uninfiltrated leaf area, and 6 days post-infiltration (6 DPI) for leaf area inoculated with the hemagglutinin (HA) expression vector. Data represent means with standard errors of the means (*n* = 3–4). In **A**, **B**, means with different lowercase letters at each *C*_a_ are significantly different by Tukey–Kramer’s HSD test at *P* < 0.05.

In Exp. 3, *P*_n_ in pre-infiltrated leaves, uninfiltrated leaves, and HA vector-inoculated leaves was measured at -1, 7, and 6 DPI, respectively, under low (250 μmol m^-2^ s^-1^) and high (1,200 μmol m^-2^ s^-1^) PPFD at various *C*_a_ levels. We previously found that leaf HA content reached the maximum level at approximately 6 DPI under the growth conditions similar to those in the present experiment (Matsuda et al., 2017a). Pre-infiltrated and uninfiltrated leaves exhibited comparable *C*_a_–*P*_n_ curves under both PPFD levels (**Figures 3A,B**). At any *C*_a_ level, *P*_n_ of HA vector-inoculated leaves was significantly lower than that of pre-infiltrated and uninfiltrated leaves. To evaluate *P*_n_ excluding the effect of lower *g*_s_ in HA vector-inoculated leaves (**Figures 1C**, **2C**), *P*_n_ was plotted against *C*_i_ instead of *C*_a_ (**Figures 3C,D**). *P*_n_ at a given *C*_i_ was lower in HA vector-inoculated leaves than in pre-infiltrated and uninfiltrated leaves under both PPFD conditions. Therefore, the lower *P*_n_ in HA vector-inoculated leaves was not solely accounted for by the lower *g*_s_, and the downregulation of photosynthetic reactions in chloroplasts should occur. We also measured *F*_v_/*F*_m_ and *Y*_II_ at low and high PPFD, and they were all significantly lower in HA vector-inoculated leaves than in pre-infiltrated and uninfiltrated leaves (**Table 1**). This indicates that photosynthetic electron transport capacity of PSII was downregulated in HA vector-inoculated leaves.

**Table 1:** Maximum quantum yields of photosystem II in the dark (*F*_v_/*F*_m_) and effective quantum yields of photosystem II (*Y*_II_) at photosynthetic photon flux densities (PPFDs) of 250 and 1,200 μmol m^-2^ s^-1^ in *Nicotiana benthamiana* leaves at different days post-infiltration (DPI).

DPI	Treatment	*F*_v_/*F*_m_	*Y*_II_	
			PPFD (μmol m^-2^ s^-1^)	
			250	1,200
-1	Pre-infiltrated	0.81 ± 0.004a	0.72 ± 0.005a	0.51 ± 0.012a
7	Uninfiltrated	0.81 ± 0.006a	0.72 ± 0.004a	0.47 ± 0.016a
6	HA	0.76 ± 0.014b	0.63 ± 0.015b	0.34 ± 0.015b

*Means ± standard errors of the means (*n* = 3–4). Means with different lowercase letters in each column are significantly different by Tukey–Kramer’s HSD test at *P* < 0.05*.

### Plant Growth

We next confirmed the effect of reduction of leaf *P*_n_ in leaves inoculated with the empty and HA vectors on plant growth. Plants that had been inoculated with either of the vectors or uninfiltrated were grown and harvested at 6 DPI, and leaf DM was compared (**Table 2**). There was no significant difference in leaf DM among them, although their leaf DM was significantly greater than that pre-infiltration. Thus, the effect of leaf *P*_n_ reduction due to the vector inoculation on plant growth was not clearly observed under the conditions of the present study.

**Table 2:** Leaf dry mass of *Nicotiana benthamiana* plants at 0 and 6 days post-infiltration (DPI).

DPI	Treatment	Leaf dry mass (g)
0	Pre-infiltrated	1.09 ± 0.155b
6	Uninfiltrated	1.51 ± 0.087a
6	Empty vector	1.57 ± 0.177a
6	HA vector	1.83 ± 0.131a

*Means ± standard errors of the means (*n* = 3). Means with different lowercase letters are significantly different by Tukey–Kramer’s HSD test at *P* < 0.05*.

### Changes in Hemagglutinin Content and Leaf Temperature at Different Air Temperatures

**FIGURE 4:**
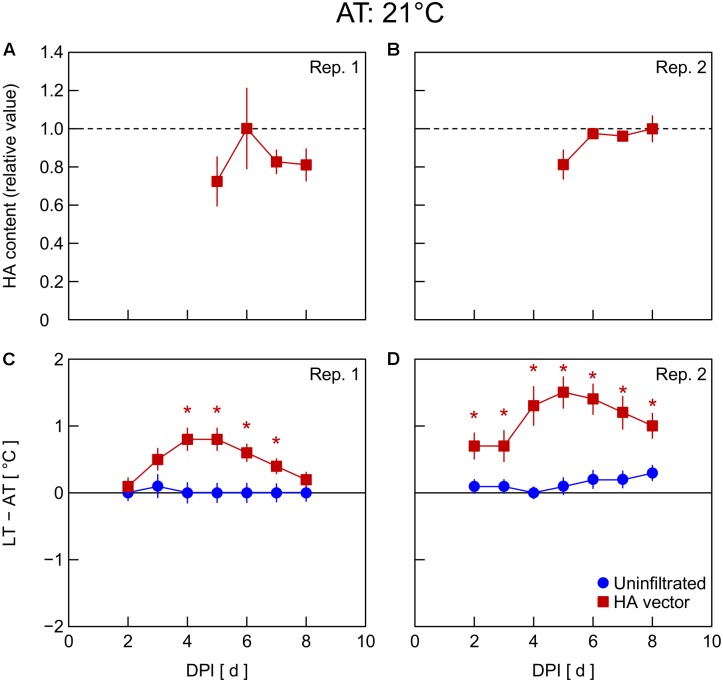
Time course of relative hemagglutinin (HA) content per unit dry mass **(A,B)** and leaf and air temperature difference (LT – AT, **C,D**) in leaves of *Nicotiana benthamiana* plants grown at an AT of 21°C at different days post-infiltration (DPI) (Exp. 4). Plants were uninfiltrated or inoculated with the hemagglutinin (HA) expression vector. Experiments were replicated twice. Data represent means with standard errors of the means (*n* = 4 for **A,B**; 6 for **C,D**). An asterisk (^∗^) represents a significant difference between uninfiltrated leaves and HA vector-inoculated leaves at each DPI by Welch’s *t*-test at *P* < 0.05.

**FIGURE 5:**
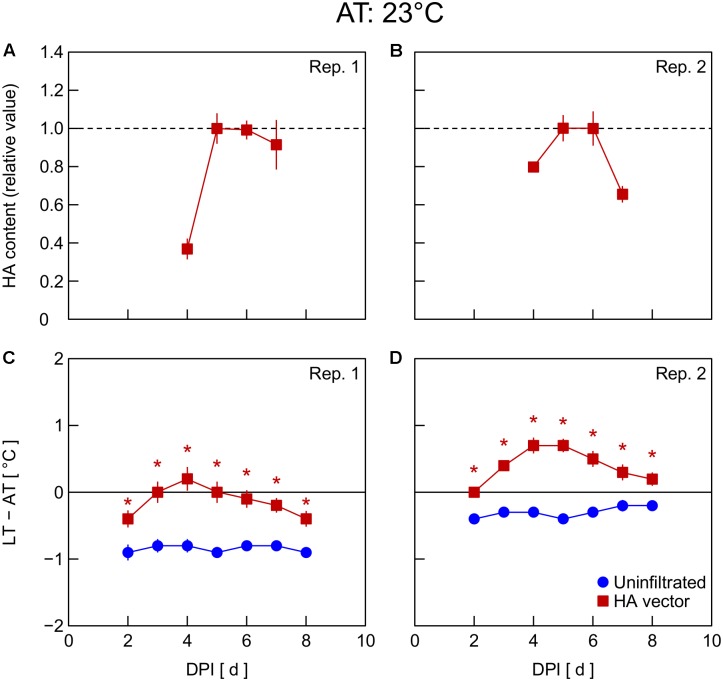
Time course of relative hemagglutinin (HA) content per unit dry mass **(A,B)** and leaf and air temperature difference (LT – AT, **C,D**) in leaves of *Nicotiana benthamiana* plants grown at an AT of 23°C at different days post-infiltration (DPI) (Exp. 4). Plants were uninfiltrated or inoculated with the hemagglutinin (HA) expression vector. Experiments were replicated twice. Data represent means with standard errors of the means (*n* = 4 for **A,B**; 6 for **C,D**). An asterisk (^∗^) represents a significant difference between uninfiltrated leaves and HA vector-inoculated leaves at each DPI by Welch’s *t* test at *P* < 0.05.

**FIGURE 6:**
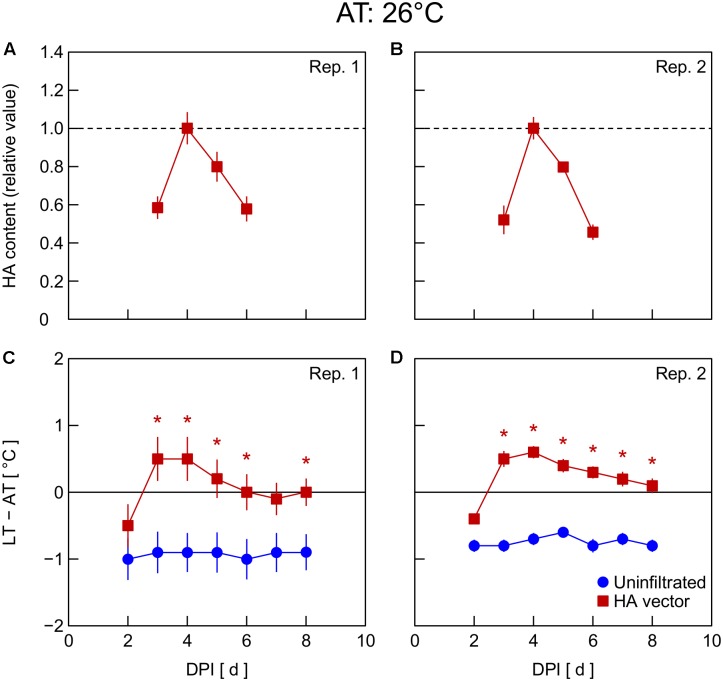
Time course of relative hemagglutinin (HA) content per unit dry mass **(A,B)** and leaf and air temperature difference (LT – AT, **C,D**) in leaves of *Nicotiana benthamiana* plants grown at an AT of 26°C at different days post-infiltration (DPI) (Exp. 4). Plants were uninfiltrated or inoculated with the hemagglutinin (HA) expression vector. Experiments were replicated twice. Data represent means with standard errors of the means (*n* = 4 for **A,B**; 6 for **C,D**). An asterisk (^∗^) represents a significant difference between uninfiltrated leaves and HA vector-inoculated leaves at each DPI by Welch’s *t* test at *P* < 0.05.

In Exp. 4, changes in HA content and LT – AT in leaves were examined at different AT levels post-infiltration. At 21°C AT, HA content increased from 5 to 6 DPI and thereafter decreased slightly (**Figure 4A**) or was almost unchanged (**Figure 4B**). At 23°C AT, HA content increased from 4 to 5 DPI, attained high levels at 5 and 6 DPI, and decreased from 6 to 7 DPI (**Figures 5A,B**). At 26°C AT, HA content increased from 3 to 4 DPI and decreased sharply from 4 to 6 DPI (**Figures 6A,B**). Thus, the greater the post-infiltration AT, the earlier the accumulation of HA in leaves and the timing at which HA content peaked.

The LT – AT during the overall experimental period was higher as the AT became higher, both in uninfiltrated and HA vector-inoculated leaves (**Figures 4–6C,D**). In uninfiltrated leaves, LT – AT was almost unchanged throughout the period irrespective of AT. In contrast, in HA vector-inoculated leaves, LT – AT first increased, attained its peak, and then gradually decreased for all AT levels. Furthermore, the DPI that was required for LT – AT to reach the maximum level appeared to decrease with increasing AT, i.e., it was approximately 5, 4, and 3–4 DPI at 21, 23, and 26°C, respectively.

## Discussion

### Gas Exchange Rates Are Reduced and Photosynthetic Capacity Is Downregulated in Leaves Transiently Overexpressing Hemagglutinin With the Viral Vector

Inoculation of *N. benthamiana* leaves with the HA expression vector significantly decreased *P*_n_ and *T* from 2 to 5 DPI compared to uninfiltrated leaves (**Figures 1A,B**) and leaves that were vacuum infiltrated with the mock *A. tumefaciens* suspension and did not receive a T-DNA vector (**Figures 2A,B**). Inoculation of leaves with the empty vector not containing the sequence of HA gene decreased *P*_n_ (**Figures 1**, **2A**) and *T* (**Figures 1**, **2B**) yet not as much as did inoculation with the HA expression vector. Thus, the viral vector inoculation itself decreased *P*_n_ and *T* in leaves, and the HA expression further decreased them. Moreover, the difference in *g*_s_ among treatments showed a similar trend to those observed in *P*_n_ and *T* (**Figures 1**, **2**), suggesting that the decreases in *P*_n_ and *T* in empty vector- and HA vector-inoculated leaves were at least partly accounted for by the decrease in *g*_s_. Infection of pathogens including viruses generally leads to reduction of *g*_s_ (Grimmer et al., 2012). Salicylic acid accumulation during the hypersensitive response is thought to be involved in the reduction of *g*_s_ via stomatal closure (Chaerle et al., 1999). Such response to virus infection might at least partly explain what was observed in the viral vector-inoculated leaves during the present study. Hamorsky et al. (2015) reported that inoculating *N. benthamiana* leaves with the same empty vector as used in the present study and with vectors to express cholera toxin B subunit resulted in a greater than 100-fold increase in the expression of *PR1a*, a marker gene for the hypersensitive response, compared to uninfiltrated leaves.

According to a biochemical model of C_3_ photosynthesis, the so-called FvCB model (Farquhar et al., 1980), under high PPFD conditions, *P*_n_ measured at low and high *C*_i_ is limited by the capacity of RuBisCO to assimilate CO_2_ and the capacity of photosynthetic electron transport to regenerate ribulose-1,5-bisphosphate in the Calvin cycle, respectively. Under low PPFD conditions, *P*_n_ is limited by the electron transport capacity including light harvesting. *P*_n_ in HA vector-inoculated leaves was lower than that in uninfiltrated leaves irrespective of PPFD or *C*_i_ (**Figures 3C,D**), suggesting that both capacities of CO_2_ assimilation by RuBisCO and electron transport were downregulated *in planta*. In previous studies, the apparent reduction of RuBisCO content in leaves transiently overexpressing a foreign protein with viral vectors has been observed (Marillonnet et al., 2004; Matsuda et al., 2012). This might be related to the remarkable reduction of *P*_n_ in HA vector-inoculated leaves. In addition, results of our fluorometric analysis (**Table 1**) suggest that components involved in the photosynthetic electron transport chain were also downregulated in HA vector-inoculated leaves. It is likely that transient HA overexpression with the viral vector downregulated overall photosynthetic machinery in HA vector-inoculated leaves. Thus, both the decrease in *g*_s_ and the downregulation of photosynthesis in mesophyll cells should contribute to the decreased *P*_n_ by the HA expression in *N. benthamiana* leaves.

Despite the significant decrease in *P*_n_ in empty vector- and HA vector-inoculated plants, their leaf DM at 6 DPI was not significantly different from that of the uninfiltrated control (**Table 2**). This was probably because the post-infiltration period was only for 6 days and therefore the integral of the decreased *P*_n_ in the viral vector-inoculated leaves during that period was too small to appear as a reduction in leaf DM. However, it is possible that downregulation of photosynthesis negatively affects metabolisms in plant cells via a supply shortage of energy and substrates. Specification of such negative effects, if any, will be a future research subject.

### Leaf Temperature Increases Prior to Hemagglutinin Accumulation

Leaf temperature increased post-infiltration in empty vector- and HA vector-inoculated leaves, whereas LT was almost constant in uninfiltrated leaves and leaves infiltrated with the mock suspension (**Figures 1**, **2D**). It is likely that the decrease in *T* (**Figures 1**, **2B**) accompanied by the decrease in *g*_s_ (**Figures 1**, **2C**) in vector-inoculated leaves primarily contributed to the increase in LT via the suppression of latent heat dissipation. Chaerle et al. (1999) observed an increase in LT due to stomatal closure in *N. tabacum* leaves infected by the tobacco mosaic virus. Such response to virus infection might explain what was observed in the viral vector-inoculated leaves in the present study.

Temperature is known as an environmental factor that significantly influences the accumulation of various recombinant proteins in plants in transient gene expression systems (for a review, see Fujiuchi et al., 2016a). When AT post-infiltration was maintained at 26, 23, and 21°C, the highest levels of leaf HA content were observed at 4 DPI (**Figures 6C,D**), 5–6 DPI (**Figures 5C,D**), and 6 DPI and later (**Figures 4C,D**), respectively. We previously reported a similar temperature-dependent difference in the time course of leaf HA content (Matsuda et al., 2017a). The activity of RNA replicase of tobacco mosaic virus almost linearly increases with increasing temperature between 12 and 35°C (White and Dawson, 1978), which might be a factor contributing to the early accumulation of HA at high AT. Peak LT was also attained earlier as post-infiltration AT increases (**Figures 4–6C,D**). We recently reported that mean LT rather than mean AT post-infiltration was better at accounting for temperature dependency of the leaf HA level at 6 DPI under various thermal conditions and identified the importance of LT post-infiltration (Matsuda et al., 2017b). Based on our results in the present study, the time course of LT as well as mean LT post-infiltration should be considered when evaluating the effect of temperature on recombinant protein accumulation.

The peak LT was attained 1–2 days earlier than the peak HA content irrespective of AT. This finding leads us to propose the possibility of using thermal information of leaves for the non-destructive estimation of an appropriate DPI of harvest at which time the leaf HA content reaches almost its maximum level at each AT. For example, the day at which time comparable LT to or lower LT than that on the day before was detected can be appropriate for harvesting to obtain a high HA yield. AT can vary spatiotemporally in a greenhouse or even in a fully contained, environmentally controlled facility used for post-infiltration plant cultivation. If LT is monitored with infrared thermography, for example, the optimum harvest date, which might vary depending on individual plants due to the AT variation, could be detected. Indeed, Buyel et al. (2016) proposed usefulness of a non-invasive contact-free method to determine thermal properties of leaves in plant-based biopharmaceutical protein production to meet a prerequisite for good manufacturing practice, i.e., monitoring plant growth, maturation, and batch-to-batch consistency. Although application of this concept to practical production systems remains to be demonstrated, the present study presents the possibility of introducing a non-invasive sensing technique into the management of the upstream plant production process in viral vector-based biopharmaceutical protein production systems.

## Conclusion

Viral vector inoculation significantly reduced *P*_n_ and *T* in *N. benthamiana* leaves, which was associated with the reduction of *g*_s_. Inoculation of leaves with the empty vector itself decreased *P*_n_, *T*, and *g*_s_ to some extent and the HA expression further decreased them. In HA vector-inoculated leaves, downregulation of photosynthetic capacity including electron transport activity occurred, in addition to the impairment of stomatal opening. LT increased in viral vector-inoculated leaves, which was associated with a decrease in *T*. HA accumulated earlier in leaves as AT post-infiltration was increased, and the peak LT appeared to be attained 1–2 days earlier than the peak HA content irrespective of AT. This suggests a possibility to use LT information to determine harvest date of leaves, at which time leaf HA content was nearly maximum, without destructive measurements.

## Author Contributions

RM conceived and designed the study. AU and HN performed the experiments. All authors analyzed the data and reviewed the manuscript. RM wrote the manuscript.

## Conflict of Interest Statement

The authors declare that the research was conducted in the absence of any commercial or financial relationships that could be construed as a potential conflict of interest.

## Footnotes

1 http://medicago.com

## References

[B1] Bally J. Nadai M. Vitel M. Rolland A. Dumain R. Dubald M. (2009). Plant physiological adaptations to the massive foreign protein synthesis occurring in recombinant chloroplasts. *Plant Physiol.* 150 1474–1481. 10.1104/pp.109.139816 19458113 PMC2705049

[B2] Buyel J. F. Gruchow H. M. Tödter N. Wehner M. (2016). Determination of the thermal properties of leaves by non-invasive contact-free laser probing. *J. Biotechnol.* 217 100–108. 10.1016/j.jbiotec.2015.11.008 26608794

[B3] Chaerle L. Van Caeneghem W. Messens E. Lambers H. Van Montagu M. Van Der Straeten D. (1999). Presymptomatic visualization of plant–virus interactions by thermography. *Nat. Biotechnol.* 17 813–816. 10.1038/11765 10429250

[B4] Chaerle L. Van Der Straeten D. (2001). Seeing is believing: imaging techniques to monitor plant health. *Biochim. Biophys. Acta* 1519 153–166. 10.1016/S0167-4781(01)00238-X 11418181

[B5] Chen Q. Lai H. Hurtado J. Stahnke J. Leuzinger K. Dent M. (2013). Agroinfiltration as an effective and scalable strategy of gene delivery for production of pharmaceutical proteins. *Adv. Tech. Biol. Med.* 1:103. 10.4172/atbm.1000103 25077181 PMC4113218

[B6] Desai P. N. Shrivastava N. Padh H. (2010). Production of heterologous proteins in plants: strategies for optimal expression. *Biotechnol. Adv.* 28 427–435. 10.1016/j.biotechadv.2010.01.005 20152894

[B7] Drake P. M. W. Szeto T. H. Paul M. J. Teh A. Y.-H. Ma J. K.-C. (2017). Recombinant biologic products versus nutraceuticals from plants – a regulatory choice? *Br. J. Clin. Pharmacol.* 83 82–87. 10.1111/bcp.13041 27297459 PMC5338133

[B8] Farquhar G. D. von Caemmerer S. Berry J. A. (1980). A biochemical model of photosynthetic CO2 assimilation in leaves of C3 species. *Planta* 149 78–90. 10.1007/BF00386231 24306196

[B9] Fischer R. Emans N. (2000). Molecular farming of pharmaceutical proteins. *Transgenic Res.* 9 279–299. 10.1023/A:1008975123362 11131007

[B10] Fischer R. Schillberg S. Hellwig S. Twyman R. M. Drossard J. (2012). GMP issues for recombinant plant-derived pharmaceutical proteins. *Biotechnol. Adv.* 30 434–439. 10.1016/j.biotechadv.2011.08.007 21856403

[B11] Fu Z. Q. Dong X. (2013). Systemic acquired resistance: turning local infection into global defense. *Annu. Rev. Plant Biol.* 64 839–863. 10.1146/annurev-arplant-042811-105606 23373699

[B12] Fujiuchi N. Matoba N. Matsuda R. (2016a). Environment control to improve recombinant protein yields in plants based on *Agrobacterium*-mediated transient gene expression. *Front. Bioeng. Biotechnol.* 4:23. 10.3389/fbioe.2016.00023 27014686 PMC4781840

[B13] Fujiuchi N. Matsuda R. Matoba N. Fujiwara K. (2016b). Removal of bacterial suspension water occupying the intercellular space of detached leaves after agroinfiltration improves the yield of recombinant hemagglutinin in a *Nicotiana benthamiana* transient gene expression system. *Biotechnol. Bioeng.* 113 901–906. 10.1002/bit.25854 26461274

[B14] Genty B. Briantais J.-M. Baker N. R. (1989). The relationship between the quantum yield of photosynthetic electron transport and quenching of chlorophyll fluorescence. *Biochim. Biophys. Acta* 990 87–92. 10.1016/S0304-4165(89)80016-9

[B15] Gleba Y. Klimyuk V. Marillonnet S. (2007). Viral vectors for the expression of proteins in plants. *Curr. Opin. Biotechnol.* 18 134–141. 10.1016/j.copbio.2007.03.002 17368018

[B16] Gleba Y. Marillonnet S. Klimyuk V. (2004). Engineering viral expression vectors for plants: the ‘full virus’ and the ‘deconstructed virus’ strategies. *Curr. Opin. Plant Biol.* 7 182–188. 10.1016/j.pbi.2004.01.003 15003219

[B17] Grimmer M. K. Foulkes M. J. Paveley N. D. (2012). Foliar pathogenesis and plant water relations: a review. *J. Exp. Bot.* 63 4321–4331. 10.1093/jxb/ers143 22664583

[B18] Hamorsky K. T. Kouokam J. C. Jurkiewicz J. M. Nelson B. Moore L. J. Husk A. S. (2015). N-Glycosylation of cholera toxin B subunit in *Nicotiana benthamiana*: impacts on host stress response, production yield and vaccine potential. *Sci. Rep.* 5:8003. 10.1038/srep08003 25614217 PMC4303877

[B19] Kitajima M. Butler W. L. (1975). Quenching of chlorophyll fluorescence and primary photochemistry in chloroplasts by dibromothymoquinone. *Biochim. Biophys. Acta* 376 105–115. 10.1016/0005-2728(75)90209-1 1125215

[B20] Koncz C. Schell J. (1986). The promoter of T_L_-DNA gene *5* controls the tissue-specific expression of chimaeric genes carried by a novel type of *Agrobacterium* binary vector. *Mol. Gen. Genet.* 204 383–396. 10.1007/BF00331014

[B21] Lam E. Kato N. Lawton M. (2001). Programmed cell death, mitochondria and the plant hypersensitive response. *Nature* 411 848–853. 10.1038/35081184 11459068

[B22] Lico C. Chen Q. Santi L. (2008). Viral vectors for production of recombinant proteins in plants. *J. Cell Physiol.* 216 366–377. 10.1002/jcp.21423 18330886 PMC7166642

[B23] Ma J. K.-C. Drake P. M. W. Christou P. (2003). The production of recombinant pharmaceutical proteins in plants. *Nat. Rev. Genet.* 4 794–805. 10.1038/nrg1177 14526375

[B24] Mandadi K. K. Scholthof K. B. G. (2013). Plant immune responses against viruses: how does a virus cause disease? *Plant Cell* 25 1489–1505. 10.1105/tpc.113.111658 23709626 PMC3694688

[B25] Marillonnet S. Giritch A. Gils M. Kandzia R. Klimyuk V. Gleba Y. (2004). *In planta* engineering of viral RNA replicons: efficient assembly by recombination of DNA modules delivered by *Agrobacterium*. *Proc. Natl. Acad. Sci. U.S.A.* 101 6852–6857. 10.1073/pnas.0400149101 15103020 PMC406431

[B26] Marillonnet S. Thoeringer C. Kandzia R. Klimyuk V. Gleba Y. (2005). Systemic *Agrobacterium tumefaciens*-mediated transfection of viral replicons for efficient transient expression in plants. *Nat. Biotechnol.* 23 718–723. 10.1038/nbt1094 15883585

[B27] Matoba N. Davis K. R. Palmer K. E. (2011). Recombinant protein expression in *Nicotiana*. *Methods Mol. Biol.* 701 199–219. 10.1007/978-1-61737-957-4_11 21181532

[B28] Matsuda R. Abe T. Fujiuchi N. Matoba N. Fujiwara K. (2017a). Effect of temperature post viral vector inoculation on the amount of hemagglutinin transiently expressed in *Nicotiana benthamiana* leaves. *J. Biosci. Bioeng.* 124 346–350. 10.1016/j.jbiosc.2017.04.007 28460871

[B29] Matsuda R. Abe T. Fujiwara K. (2017b). Viral vector-based transient gene expression in *Nicotiana benthamiana*: effects of light source on leaf temperature and hemagglutinin content. *Plant Cell Rep.* 36 1667–1669. 10.1007/s00299-017-2164-6 28608187

[B30] Matsuda R. Kushibiki T. Fujiuchi N. Fujiwara K. (2018). Agroinfiltration of leaves for deconstructed viral vector-based transient gene expression: infiltrated leaf area affects recombinant hemagglutinin yield. *Hortic. Environ. Biotechnol.* 59 547–555.

[B31] Matsuda R. Tahara A. Matoba N. Fujiwara K. (2012). Virus-vector mediated rapid protein production in *Nicotiana benthamiana*: effects of temperature and photosynthetic photon flux density on hemagglutinin accumulation. *Environ. Control Biol.* 50 375–381. 10.2525/ecb.50.375

[B32] Peyret H. Lomonossoff G. P. (2015). When plant virology met *Agrobacterium*: the rise of the deconstructed clones. *Plant Biotechnol. J.* 13 1121–1135. 10.1111/pbi.12412 26073158 PMC4744784

[B33] Pogue G. P. Lindbo J. A. Garger S. J. Fitzmaurice W. P. (2002). Making an ally from an enemy: plant virology and the new agriculture. *Annu. Rev. Phytopathol.* 40 45–74. 10.1146/annurev.phyto.40.021102.150133 12147754

[B34] Pogue G. P. Vojdani F. Palmer K. E. Hiatt E. Hume S. Phelps J. (2010). Production of pharmaceutical-grade recombinant aprotinin and a monoclonal antibody product using plant-based transient expression systems. *Plant Biotechnol. J.* 8 638–654. 10.1111/j.1467-7652.2009.00495.x 20514694

[B35] R Core Team (2017). *R: A Language and Environment for Statistical Computing*. Vienna: R Foundation for Statistical Computing.

[B36] Rolfe S. A. Scholes J. D. (2010). Chlorophyll fluorescence imaging of plant–pathogen interactions. *Protoplasma* 247 163–175. 10.1007/s00709-010-0203-z 20814703

[B37] Roychowdhury S. Oh Y. J. Kajiura H. Hamorsky K. T. Fujiyama K. Matoba N. (2018). Hydroponic treatment of *Nicotiana benthamiana* with kifunensine modifies the N-glycans of recombinant glycoprotein antigens to predominantly Man9 high-mannose type upon transient overexpression. *Front. Plant Sci.* 9:62. 10.3389/fpls.2018.00062 29441088 PMC5797603

[B38] Twyman R. M. Stoger E. Schillberg S. Christou P. Fischer R. (2003). Molecular farming in plants: host systems and expression technology. *Trends Biotechnol.* 21 570–578. 10.1016/j.tibtech.2003.10.002 14624867

[B39] von Caemmerer S. Farquhar G. D. (1981). Some relationships between the biochemistry of photosynthesis and the gas exchange of leaves. *Planta* 153 376–387. 10.1007/BF00384257 24276943

[B40] White J. L. Dawson W. O. (1978). Effect of supraoptimal temperatures upon tobacco mosaic virus RNA replicase. *Intervirology* 10 221–227. 10.1159/000148985 681145

